# Policies for Material Circularity: the Case of Lithium

**DOI:** 10.1007/s43615-022-00171-z

**Published:** 2022-05-17

**Authors:** Diana Roa, Knut Einar Rosendahl

**Affiliations:** Norwegian University of Life of Sciences, Ås, Norway

**Keywords:** Critical raw material, Lithium, Recycling subsidy, Disposal charge, Material rebound, Dynamic optimization

## Abstract

Improper waste management carries social risks and dissipates high-value materials. Moreover, material market prices do not reflect these hidden costs and values. Two important questions are how prices can inform society about their resource use impact and how market-based policies optimize material circularity. This study adds to the literature by analyzing the effect of market-based policies aimed at promoting circular material reuse in a market defied by harmful waste but enhanced by recycling. The findings indicate that a landfill tax is a first-best policy since it targets the external costs of waste disposal, improves welfare, reduces damages, and boosts recycling. If a landfill tax is not feasible, other programs like taxes, subsidies, and a tax-subsidy scheme provide second-best results. Remarkably, recycling subsidies can stimulate higher raw material extraction and generate rebound effects. We also explore other non-market-based strategies to prevent waste and make recycling more cost-competitive and easier to recycle. The numerical results and sensitivity analysis of the lithium market illustrate the model's flexibility and prove why some policies are superior to others for reducing waste and creating value from used materials. Our study results serve as a guide to designing policies for optimal material circularity.

## Introduction

Material efficiency is crucial to support the transition towards a low-carbon, digital economy. Electronic devices and emerging technologies like electric vehicles and smart grid batteries require vast raw materials. A primary concern is that scarcity and supply risks may threaten to slow down the green and digital transitions [1–3]. However, recent research reveals that the rising demand for electronics is causing a surge in electronic waste (e-waste) [4]. To prevent social risks and limit valuable material losses, society must dispose of waste safely. Otherwise, as an environmental externality, waste impairs welfare and sustainability. Therefore, improper e-waste management undermines the promising benefits of the digital revolution and green shift.

This challenge calls for policy intervention. As a rule, waste management policy incentives upstream and downstream spheres [5–8]. Upstream refers to products designed with the environment in mind, and downstream aims at efficient recycling, diverting waste from landfills.^1^ Fullerton and Wu [6] analyzed downstream policies and found that charging consumers the marginal social costs of disposal can correct the market failure and even persuade firms to design more recyclable products. Although their theoretical analysis proves how recyclability affects consumers’ utility levels, in practice, consumers may be willing to recycle, but it will depend on better-organized recycling and collection systems [10, 11].

Recycling offers a way to manage harmful waste and exploit long-lasting materials once it becomes an attractive market. Unfortunately, recycling e-waste is seldom profitable due to costly and nascent reprocessing technologies compared to cheap and mature mining [12]. However, even unprofitable recycling can improve welfare when market prices do not reflect externalities [13]. A common government practice is subsidizing private recyclers to undertake risks and reduce cost pressures. Although research has backed that idea [14, 15] and suggests governments invest in research and improve technologies to make recycling more operational [16], there may be fiscal constraints that question to what extent recycling subsidies are the preferred policy compared to other measures.

The discussion so far highlights the difficulty of promoting policies that, in unison, steer producers, consumers, and recyclers’ behavior and sustainably reorient public finances. Research suggests no single tool can solve multiple problems simultaneously, such as promoting recycling and reducing waste and damage [5–7, 17]. Some researchers find that disposal fees are insufficient without regulatory measures to ensure better product design [8]. Other studies argue that a tax-subsidy scheme can correct market failures related to waste disposal [5, 18]. In other cases, combining taxes on raw materials with subsidies for recycling does not work well due to distorting effects from the recycling subsidy [17]. Thus far, research has focused on optimizing inefficient markets and overemphasized recycling as a means of reducing material scarcity [19]. However, there is still a lack of thorough exploration of the cumulative effects of waste management policies on welfare and damages considering budget constraints. This paper, therefore, provides a quantitative analysis that complements previous mostly theoretical studies on waste management.

The term circular reuse throughout this paper implies reducing e-waste to a minimum and creating added value from used materials. With that in mind, this study cannot cover all environmental impacts at different stages of a materials’ life cycle, as we are not looking at the environmental externalities of mining in ecologically sensitive areas or carbon emissions from material recycling. This study focuses only on end-of-life product externalities. Therefore, our policy analysis is strongly Pigouvian based on the user or polluter pays principle to internalize externalities from waste disposal, which can also stimulate material efficiency.

Our study aims to analyze the impact of market-based policies to promote material circular reuse in a market enhanced by recycling and defied by hazardous waste. We ask in this paper how prices can inform society about their resource use impact and how market-based policies can optimize material circularity. Our model incorporates a material balance condition, waste damage costs, and non-linear mining and recycling costs. By examining how producers, consumers, and recyclers behave under constraints, this study offers new insight into policy design for waste management. A first-best policy maximizes welfare and achieves efficient recycling levels to reduce waste. When that first-best is not feasible, we must rely on other policies denoted as second-best solutions. Our simulations of the lithium market^2^ and a sensitivity analysis on key assumptions illustrate the model's flexibility. Lastly, we discuss why some strategies are superior to others and examine some of the policy counterfactual effects and implementation challenges.

## Model Assumptions

Our analysis builds on the Hotelling model for non-renewable resources and introduces a material balance constraint, and non-linear extraction and recycling costs. This model extends the framework presented in Rosendahl and Rubiano [19] by including a negative externality from waste disposal. The approach uses a partial equilibrium analysis of a durable resource market to focus on two aspects: (i) the resource market equilibrium, including recycling, but disregarding interactions with other markets; (ii) the Marshallian aggregate surplus as a welfare measure to compare policies. The benefit of this method is that one can observe how the market works at suboptimal levels because prices do not reflect waste disposal costs (“Free Market Solution”). Then, by comparing the free market with the socially optimal solution (“Social Planner Solution”), we can introduce market-based policies to deal with market failures (“Market-Based Policies”).

**Fig. 1:**
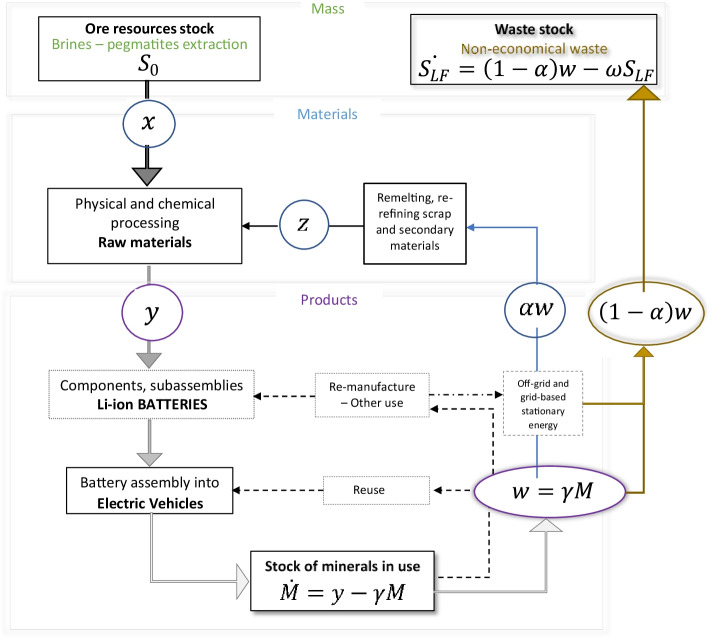
Conceptual map: squared boxes represent materials or product stocks, and circles represent flow variables. Solid lines show what is included in this paper, while dashed lines display variables out of the scope of this study. We explain the variables and parameters in detail in “Free Market Solution”

Figure 1 shows the relationship between ore resource stocks and material flows. After being mined, lithium metal becomes battery-grade material. The conceptual map below also summarizes the variables used in our model, which we measure in value terms, not physical terms. Notice that used material can be recycled and returned to the market or end up as an uneconomical waste.

### Free Market Solution

In the unregulated market solution, no one considers waste damage costs in their decisions. We assume free entry and competitive behavior in the mining and recycling sectors.^3^

Let ${P}^{M}$ denote the material market price, i.e., raw ($x$) or recycled material ($z$). Furthermore, let ${P}^{W}$ be the waste price ($w$) collected from consumers by recyclers. This price can be positive or negative (see Eq. (3)).

#### Recycling

The competitive recycling industry collects waste ($w$) from consumers at the price ${P}^{W}$. Whether the waste price ${(P}^{W})$ is positive or negative depends on recycling profitability versus the costs of delivering waste to the landfill (Eq. (3)). If landfill costs are high (e.g., due to a landfill tax), we assume that the recyclers cannot avoid this payment by throwing the waste elsewhere.^4^

An amount of recycled scrap $\left(z=\alpha w\right)$ is sold in the market, while the remaining part $\left(\left(1-\alpha \right)w\right)$ is delivered to the landfill at a price ${P}^{LF}\ge 0$. We assume that storing scrap is too costly to be profitable. Hence, recyclers do not face an intertemporal trade-off between current and future stocks, so their maximization problem is unconstrained from a stock variable. The recycling rate ($0<\alpha <1$) is endogenous, depending on recycling profitability.^5^

Recycling costs appear as ${C}^{R}\left(\alpha \right)z$, and marginal recycling costs are strictly increasing in the share of recycled output: ${C}_{\alpha }^{R}>0$ and ${C}_{\alpha \alpha }^{R}>0$. The term ${C}_{\alpha }^{R}$ can be interpreted as the long-run average unit costs and disregard economies of scale that may appear at initial recycling stages.^6^ Moreover, we assume that $\underset{\alpha \to 1}{\mathrm{lim}}{C}_{\alpha }^{R}=\infty$ indicating that complete recycling is impossible because of the limits imposed by product design, recycling technologies, and thermodynamics of separation [25]. Therefore, we always have $\alpha <1$. The recyclers’ instantaneous profit maximization problem becomes^7^:

1 $$\underset{\alpha ,w\ge 0}{\mathrm{max}}{\pi }^{R}=\left[{P}^{M}\alpha -{P}^{W}-{C}^{R}\left(\alpha \right)\alpha -\left(1-\alpha \right){P}^{LF}\right]w$$

We maximize with respect to $\alpha$ and $w$:

2 $$\alpha : {P}^{M}={C}_{\alpha }^{R}-{P}^{LF}$$

3 $$w: {P}^{W}={\alpha (P}^{M}{-C}^{R})-\left(1-\alpha \right){P}^{LF}$$

As stated in Eq. (2), recycling is zero ($$\alpha =0)$$ if the material price ($${P}^{M}$$) is too low to cover the marginal recycling cost $${(C}_{\alpha }^{R})$$ minus the private landfill cost ($${P}^{LF})$$. Therefore, recycling levels depend not only on the remaining earnings from material prices and recycling costs but also on disposal costs.

Equation (3) provides the zero-profit condition. The price ${(P}^{W})$ that clears the market for scrap materials depends on recycling profits given market prices, recycling costs, and landfill costs. Without recycling ($\alpha =0$), waste prices ${(P}^{W})$ equal landfill costs ${(-P}^{LF})$ and are hence zero or negative. If waste prices (${P}^{W}$) are higher than the right-hand side of Eq. (3), no recyclers will buy any waste, and hence, ${P}^{W}$ will drop. If waste prices are too low, it will bring excess demand for waste, and ${P}^{W}$ will increase.

We see that whether the waste price $\left({P}^{W}\right)$ is positive or negative is in general ambiguous. With high recycling profits and lower disposal fees, the waste price tends to be positive. Likewise, with low profits and high disposal fees, waste prices $\left({P}^{W}\right)$ tend to be negative. A negative waste price means that recyclers will not buy scrap materials, and consumers must pay to get rid of their depreciated materials.^8^

#### Consumers

A representative consumer chooses to demand raw ($x$) and recycled materials ($z$). Both goods are homogeneous; i.e., the recycled material is not a differentiated product from the pure material. Thus, disregarding resource storage, total consumption ($y$) should not exceed total supply, giving the following market balance condition:

4 $$y\le x+z$$

Let $U\left(y\right)$ denote the consumer’s quasi-linear utility function (gross consumer surplus of consuming material),^9^ and $MU\left({y}_{t}\right)$ the marginal utility of consuming material, i.e., $U\left({y}_{t}\right)={\int }_{0}^{{y}_{t}}MU\left(s\right)ds$. Hence, $MU\left({y}_{t}\right)$ represents the marginal willingness to pay for an additional unit of the resource.

The waste stock held by consumers and available to recyclers is defined by:

5 $$w=\gamma M$$

where $\gamma$ denotes the annual depreciation rate of resource stocks in use; thus, 1/$\gamma$ measures the resource lifetime before it is recycled or discarded. The material stock in use ${M}_{t}$ develops according to^10^:

6 $$\dot{M}=y-\gamma M$$

Consumers have no choice but to let recyclers collect their material waste, also if they must pay (${P}^{W}<0$). The representative consumer faces the following problem to maximize its net Consumer Surplus $CS$ subject to (6):

7 $$\underset{y\ge 0}{\mathit{max}}CS={\int }_{0}^{\infty }\left[U\left(y\right)-{P}^{M}y+{P}^{W}\gamma M\right]{e}^{-rt}dt$$

Now ${\varphi }^{c}$ is the shadow price of the material stock in use ($M$), which could be either positive or negative depending on the future waste price (${P}^{W}$). Thus, we have the following current-value Hamiltonian: ${\mathcal{H}}^{c}=U\left(y\right)-{P}^{M}y+{P}^{W}\gamma M+{\varphi }^{C}(y-\gamma M)$ and the necessary conditions for an interior solution ($y>0$) give:

8 $$y: MU\left(y\right)={P}^{M}-{\varphi }^{c}$$

9 $${M: \dot{\varphi }}^{c}=\left(r+\gamma \right){\varphi }^{c}-\gamma {P}^{W}$$

Equation (8) states that consumers will demand materials up until the point where their marginal utility $MU\left(y\right)$ equals the material price (${P}^{M}$) minus the shadow price of resource stocks in use (${\varphi }^{c}$). Thus, marginal utility can be either higher or lower than the market price, depending on the sign of ${\varphi }^{c}$. The dynamics of ${\dot{\varphi }}^{c}$ (Eq. (9)) depends on the discount and depreciation rates $\left(r+\upgamma \right)$, and on the future waste price adjusted by the annual depreciation rate of material stocks in use (${\gamma P}^{W}$). As time goes to infinity, we have that $\underset{t\to \infty }{\mathrm{lim}}{e}^{-rt}{\varphi }^{c}{M}_{t}=0$.

#### Mining Industry

The competitive mining industry has property rights to ore resources. They extract metal minerals and transform them into materials before selling them directly to consumers. Although lithium is non-renewable, we do not consider them a finite resource stock. Instead, we assume that unit extraction cost ${C}^{E}\left({A}_{t}\right)$ increases with accumulated extraction ${A}_{t} \left({C}_{A}^{E}>0\right),$ where accumulated extraction increases according to:

10 $$\dot{A}=x$$

Total extraction costs are then given by ${C}^{E}={C}^{E}\left({A}_{t}\right){x}_{t}$. This cost function disregards short-term capacity constraints, as we are interested in the long-run effects.^11^We apply the following cost function, which also allows for technological change$\tau$:

11 $${C}^{E}\left({A}_{t}\right)={C}_{0}{e}^{\eta {A}_{t}-{\tau }_{t}}$$

The parameter $\eta$ represents the rising cost rate as accumulated production increases. We calibrate this parameter to the initial deposit stock levels for each producer.^12^To extract material volume $x$, a firm faces the following problem, subject to (10):

12 $$\underset{x\ge 0}{\mathrm{max}}{\pi }^{E}={\int }_{0}^{\infty }\left[{P}^{M}x-{C}^{E}\left(A\right)x\right]{e}^{-rt}dt$$

The current-value Hamiltonian is: ${\mathcal{H}}_{2}={P}^{M}x-{C}^{E}\left(A\right)x-{\lambda }^{E}\left(x\right)$, where we have switched sign in front of the shadow price ${\lambda }^{E}$ so that ${\lambda }^{E}\ge 0$ represents the resource rent.^13^ Thus, the necessary conditions for an interior solution (${x}_{t}>0$) are:

13 $$x: {\lambda }^{E}={P}^{M}-{C}^{E}\left(A\right)$$

14 $$A: \dot{{\lambda }^{E}}=r{\lambda }^{E}-{C}_{A}^{E}x$$

Equation (13) states that extraction ($x$) should increase to the point where the material price equals unit extraction costs plus the resource rent. This resource rent also represents the shadow price of the resource property rights. The optimal path of the resource rent from future accessible resources ($\dot{{\lambda }^{E}})$ will grow at a pace defined by the interest rate minus the change in marginal costs as extraction accumulates (${C}_{A}^{E}=\eta {C}_{0}{e}^{\eta A-{\tau }_{t}}$).^14^ As time goes to infinity, $\underset{t\to \infty }{\mathrm{lim}}{e}^{-rt}\lambda {A}_{t}=0$.

### Social Planner Solution

Let us now turn to the welfare maximization problem. The social planner acknowledges waste impacts and seeks to correct the market failure by making explicit the costs from damaging waste into the welfare function. First, we assume that waste damage ${S}_{LF}$ increases for each unit of non-recycled depreciated waste sent to landfills $\left(1-\alpha \right)w$,^15^ and decreases at a natural degradation rate ($\omega$):

15 $$\dot{{S}_{LF}}=\left(1-\alpha \right)w-\omega {S}_{LF}$$

The monetary cost of such impact is $D\left({S}_{LF}\right)$, where $D{^{\prime}},{D}^{{^{\prime}}{^{\prime}}}\ge 0$. As explained in “Free Market Solution” above, consumers do not consider waste damages. Thus, damages may affect welfare but not individual behavior. The socially optimal solution is given by maximizing the following welfare expression related to a social discount rate $\rho$:

16 $$\underset{x,\alpha ,y,\mathit{w }\ge 0}{\mathrm{max}}\Omega ={\int }_{0}^{\infty }\left[U\left(y\right)-{c}^{E}\left(A\right)x-{C}^{R}\left(\alpha \right)\alpha w-D\left({S}_{LF}\right)\right]{e}^{-\uprho t}dt$$

An additional constraint $w=\gamma M$ accounts for the waste allocation held by consumers and available to recyclers, with shadow price $\theta$ (can be positive or negative), and the constraint $y\le x+\alpha w$ with its respective shadow price$\mu \ge 0$. Now given the constraints on stock variables$\dot{A}$,$\dot{M} , \dot{{S}_{LF}}$ with their respective shadow prices$\lambda ,\varphi ,\xi$, the current-value Hamiltonian is${\mathcal{H}}_{3}=U\left(y\right)-{C}^{E}\left(A\right)x-{C}^{R}\left(\alpha \right)\alpha w-D\left({S}_{LF}\right)-\lambda \left(x\right)+\varphi \left(y-\gamma M\right)-\upxi \left(\left(1-\alpha \right)w-\omega {S}_{LF}\right)-\theta \left(w-\gamma M\right)-\mu \left(y-x-\alpha w\right)$.

Table 1 shows the first-order conditions for the control and state variables with interior solutions $$\left(x,\alpha ,w,y>0\right)$$ and reveals the differences in prices between a private free market and a socially organized solution. A competitive and functioning market will solve those price differences and make $${P}^{M}=\mu$$ and $${P}^{W}=\theta$$. Besides the socio-environmental costs, the differences in shadow prices ($$\lambda , \varphi$$) between a free and a social market solution may also be due to differences between private ($$r)$$ and social discount rates )$$\rho )$$.^16^

**Table 1:** (A) The free market is unregulated, and no agents consider damage costs. (B) In a socially efficient solution, a social planner acknowledges material waste impact. (C) Market-based policies allow prices to change to internalize waste disposal costs fully.

		(A) Free market		(B) Socially efficient solution		(C) Market-based policies		
*Material prices*	Recycler	${P}^{M}={C}_{\alpha }^{R}-{P}^{LF}$; ${P}^{LF}=0$ or ${P}^{LF}<\xi$	(2)	$\mu ={C}_{\alpha }^{R}-\xi$	(2a)	${P}^{M}={C}_{\alpha }^{R}-\widehat{{P}^{LF},}$ with $\widehat{{P}^{LF}}=\widehat{\xi }$ ${P}^{M}+\widehat{\vartheta }={C}_{\alpha }^{R}-{P}^{LF},$ with ${P}^{LF}=0$	(2b) (2c)	Landfill tax ($\widehat{\xi }$) Recyclers’ subsidy ($\widehat{\vartheta }$)
	Consumer	${P}^{M}=MU\left(y\right)+{\varphi }^{C}$	(8)	$\mu =MU\left(y\right)+\varphi$	(8a)	$\widehat{MU\left(y\right)}={P}^{M}+{P}^{T}-\widehat{\varphi }$	(8b)	Consumer Tax ($\widehat{\varphi }$)
	Producer	${P}^{M}={\lambda }^{E}+{C}^{E}\left(A\right)$	(13)	$\mu =\lambda + {C}^{E}\left(A\right)$	(13a)	No policy (tax)		
*Scrap material price*		${P}^{W}={\alpha P}^{M}-{\alpha C}^{R}-\left(1-\alpha \right){P}^{LF}$	(3)	$\theta =\alpha \mu -{\alpha C}^{R}-\left(1-\alpha \right)\xi$	(3a)	$\widehat{{P}^{W}}={\alpha P}^{M}+\widehat{\vartheta }-{\alpha C}^{R}-\left(1-\alpha \right){P}^{LF}$, with (${P}^{LF}=0$)	(3b)	Recyclers’ subsidy ($\widehat{\vartheta }$)
*Shadow prices*	Valuable waste stock	$\dot{{\varphi }^{C}}=\left(r+\gamma \right){\varphi }^{C}-\gamma {P}^{W}$	(9)	$\dot{\varphi }=\left(\uprho +\gamma \right)\varphi -\gamma \theta$	(9a)	$\widehat{\varphi }$		Consumer tax ($\widehat{\varphi }$)
	Raw material deposits	$\dot{{\lambda }^{E}}=r{\lambda }^{E}-{C}_{A}^{E}x$	(14)	$\dot{\lambda }=\uprho \lambda -{C}_{A}^{E}x$	(14a)	No policy (tax)		
	Harmful waste stock			$\dot{\xi }=\left(\rho +\omega \right)\xi -{D}^{^{\prime}}\left({S}_{LF}\right)$	(9a)	$\widehat{\xi }$		Landfill tax

### Market-Based Policies

Before examining the government interventions to correct the market failure, it is important to recall that these are downstream measures aiming at efficient recycling to divert waste from landfills.

#### Landfill Tax

When market prices do not reflect the full external costs of waste disposal (${P}^{LF}<\xi$), there are “implicit subsidies” to material consumers at the expense of society, and the recycling share (if positive) is too low. Therefore, consumers have strong incentives to dump their waste in landfills at zero cost. Conversely, positive landfill taxes will lower the waste price ${P}^{W}$, so recyclers will be less willing to buy scrap materials, and consumers will have to spend more to get rid of depreciated materials. Thus, material demand will also decline despite the lower raw material market price. If material prices ${P}^{M}$ are too low to cover recycling and landfill costs, the waste price ${P}^{W}$ will be negative. Furthermore, only if the full cost of harmful waste disposal is internalized ($\widehat{{P}^{LF}}=\widehat{\xi })$, the efficient amount of recycling will be attained. In the numerical model, we assume that the marginal damage cost of waste is constant, ${D}^{^{\prime}}\left({S}_{LF}\right)=\delta$, in which case the shadow price of harmful waste stock is:

17 $$\widehat{\xi }=\delta \left(1+\frac{1}{\rho +\omega }\right)$$

In addition, damages grow proportionally to the amount of harmful waste ${D(S}_{LF})= \delta {S}_{LF}$ where $\delta >0$ is the damage cost per ton of harmful waste. (See Appendix 3 for more details on our damage cost estimation).

#### Tax on Material Consumption — Advance Fee

A consumer tax could correct the negative externality if consumers pay the marginal social waste disposal costs, and recycling is non-viable. The tax, however, does not incentivize recycling. Still, we consider a consumer tax as an alternative policy, examining the second-best consumer tax path (in the absence of landfill tax). The tax can curb demand for materials by increasing consumer prices. Fullerton and W. Wu [6] find that if consumers must pay total marginal social costs of disposal, they will induce firms to design products that are easier to recycle. In practice, a better collection system and better information may lead to consumers recycling [11]. In our model, consumers do not have precise information and preferences on product recyclability that affect their utility levels. Thus, battery designs are controlled neither by consumers nor by recyclers. Battery recyclability is an exogenous parameter that influences recycling costs, and battery manufacturers are not considered in this model.^17^

#### Subsidies to Recycling

The free market can facilitate recycling, but government subsidies can accelerate it [14]. In contrast with Hoogmartens et al. [17] and Ino and Masueda [13], our subsidies $\widehat{\vartheta }$ on recycling affect recycling efforts directly ($\alpha$) as the subsidy is paid per recycled unit $z$ processed, and not per unit of waste collected. Thus, subsidies are meant to stimulate waste processing rather than just collecting it for landfill disposal.

In the numerical analysis, we seek the second-best recycling subsidy path that maximizes welfare given the constraint of no landfill tax (${P}^{LF}=0$). When market prices do not reflect harmful waste costs, recycling subsidies become ineffective because it creates a rebound effect. In our model, a rebound occurs when a surge in waste prices (${P}^{W}$) reduces the cost of using materials (increasing ${\varphi }^{c}$ in Eq. (9)); then, material desirability will increase and, therefore, consumption (lower $MU\left(y\right)$ in Eq. (8)).

#### Combining Consumer Taxes and Recycling Subsidies

We also consider a fourth policy option, combining recycling subsidies and consumer taxes. This scheme is somewhat similar to a deposit-refund system when consumers who buy electronic products receive a deposit, and all or part of the deposit is later refunded when consumers return their products for reuse, recycling, or safe disposal. Producers (or retailers) may collect the deposit and repay it later. We do not model an explicit refund; instead, recycling subsidies tend to increase waste prices ${P}^{W}$ and thus give consumers an implicit refund higher or lower than the deposit. We assume that this policy is fiscally neutral, meaning that the government’s net revenue from the tax-subsidy scheme equals zero in each period. With two policy instruments available instead of just one, the welfare effects should be better, but this is not necessarily the case given the fiscal constraint.

## Numerical Case Simulation

This section elaborates a numerical case simulation to understand the difference between a free market and a social planner solution and illustrate the effects of different policy scenarios. First, we show how recycling is affected in a free market with changing resource availability (“Free Markets and Resource Availability”). Then, we offer different policy outcomes (“Policy Scenarios”), and we run a sensitivity analysis to examine regulatory guidelines, i.e., standards for extended product lifespans and safer and environment-friendly design (“Sensitivity Analysis I: Non-market-Based Policies”). Lastly, we show how changes in the damage costs impact our conclusions drawn from the model (“Sensitivity Analysis II: Lower and Higher Damage Costs”).

To calibrate the model, we use data from the global lithium market with the base year 2020 and use information from seven country suppliers (Argentina, Australia, Bolivia, Chile, China, USA, and the Rest of the World) and four main consumer sectors (electric vehicles, grid storage batteries, electronic devices, and other non-battery applications). In most sectors, material recycling is possible except for non-battery applications. The numerical optimization model was performed using GAMS 28.2.0 and adopted both mixed complementarity program (MPC) and non-linear program (NCP). (For data details, see Appendices 1, 2, and 3.)

### Free Markets and Resource Availability

Let us now consider recycling in a free market with changing resource availability. In contrast to previous studies [19, 32], we find that resource scarcity should not be the main reason to promote recycling. Resources may be limited in the short term due to environmental regulations in mining, delays in concession bidding, or trade issues.^18^ However, scarcity may not be a severe issue in the long term.^19^ The most likely scenario is that exploration activities continue expanding material stocks. As a result, producers will undertake discovery projects even at a higher cost, putting more available resources at affordable prices in the market.

**Fig. 2:**
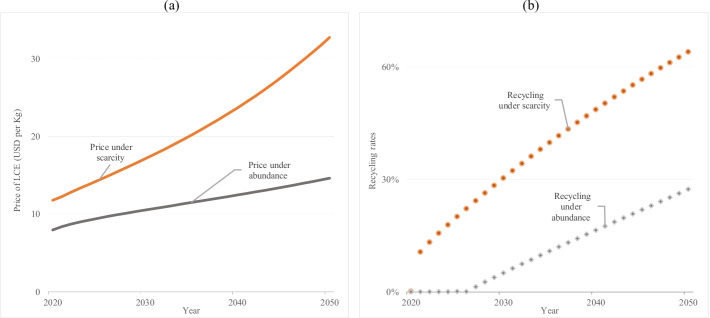
Prices, recycling rates, and resource availability

Figure 2 compares the effect on lithium market prices and recycling rates in a scarce and abundant resource scenario. In a scarcity scenario, the mining industry will exploit only economic reserves to date. When no more reserves are economically feasible, prices will range between USD 12 and 33, and recycling rates should start now at 11% to satisfy the swelling material demand. In contrast, in an abundance scenario, the mining industry can extract all identified resources; prices will range between USD 8 and 14 during the next 30 years, and recycling will not happen before 2027. Based on our estimates, exploration activity will likely expand material stocks, and without any public intervention, the market will determine very late when recycling becomes profitable regardless of harmful waste impacts.

### Policy Scenarios

This section elaborates four policy scenarios and explains the effects of optimal and suboptimal solutions over material prices, recycling rates, waste, demand, supply, welfare, and damage levels. Market prices do not reflect waste’s external costs and value in our benchmark scenario, and there is no policy intervention. Table 2 summarizes the four market-based policies presented in “Market-Based Policies” above.

**Table 2:** Policy scenarios

Policy scenario	Description	Symbol
Landfill tax	Pigouvian tax on landfill disposal (optimal solution, 1st best)	$\delta$> 0
Consumption tax	Tax on material consumption (suboptimal solution, 2nd best)	$\widehat{\varphi }$> 0
Recycling subsidy	Subsidy to recycling (suboptimal solution, 2nd best)	$\widehat{\vartheta }$> 0
Combining tax and subsidy	Tax on material consumption and subsidy to recycling, with subsidy payment not exceeding tax income for each sector (suboptimal solution, 2nd best)	$\widehat{\varphi }$> 0 and $\widehat{\vartheta }$ > 0

#### Prices

**Fig. 3:**
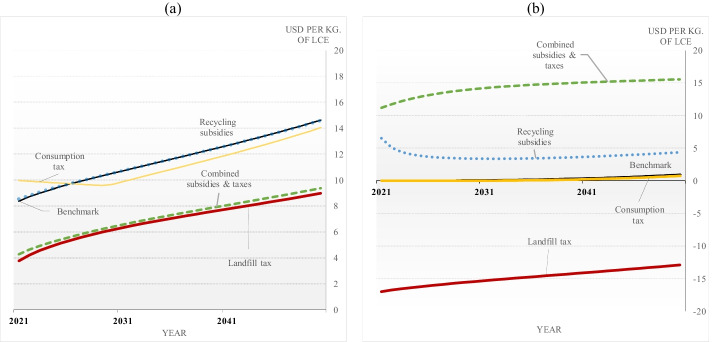
**a **Raw material market price (${P}^{M}$). **b** Waste market price (${P}^{W}$)

**Fig. 4:**
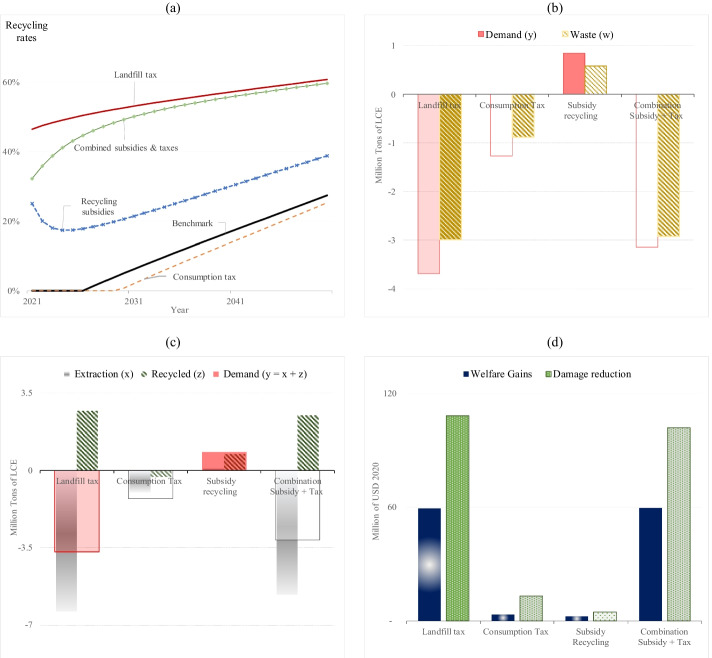
**a** The recycling rates among policy scenarios compared to the benchmark or unregulated market. **b** The difference of accumulated demand and waste (2021–2040) among policies to the benchmark. **c** The difference in raw and recycled material production (accumulated until 2040) compared to the benchmark. **d** The welfare gains and damage reduction compared to the benchmark. Here, we calculate welfare and damages over a 100-year full-time horizon

The effect of market-based policies on material prices is shown in Fig. 3a. After implementing a landfill tax, material prices attain lower levels, reducing producers’ incentives to extract lithium. The landfill tax cumulative effect on material extraction is presented in Fig. 4c.

Figure 3a shows that lithium prices also decrease after applying consumption taxes, but consumers’ purchase price (including the tax) increases. Hence, production and consumption are slightly depressed. Instead, recycling subsidies would cause the lithium market price to be close to the benchmark scenario. This effect may seem surprising at first, as an increased supply of recycled lithium would decrease lithium’s market price. However, recycling subsidies also stimulate demand for lithium waste, increasing the waste price further increasing material demand. Thus, recycling subsidies encourage both supply and demand. This situation is illustrated in Fig. 4c.

Figure 3b shows that a positive landfill tax makes the waste price negative, meaning that recyclers will not be willing to buy scrap materials, and consumers must instead pay to get rid of their depreciated materials. As a result, material demand will also diminish despite the lower market price of raw materials. By contrast, recycling subsidies make the waste price positive, further increasing material demand as consumers find materials more valuable. But this situation only occurs when recycling is profitable and delivering non-recycled waste to the landfill has low or zero cost.

Under a tax-subsidy scheme, the market price declines, while the material waste price is highest among all scenarios. As a result, recyclers deliver much more output, and the greater consumption of recycled material compensates for lower raw material demand.^20^

#### Recycling Rates

The effect of market-based policies on recycling rates is shown in Fig. 4a. It shows that after a landfill tax is in place, recycling starts immediately, and recycling rates are consistently at much higher levels than in the benchmark because recyclers can reduce the pressure of additional tariffs by increasing the amount of waste recycled and, consequently, reducing the amount of waste sent to landfills. Therefore, a disposal fee provides higher incentives to recycle.

What stands out in Fig. 4a is that after applying subsidies, recyclers hardly alter their output, and a large amount of waste ends up in the landfill despite the subsidy (after possibly being recycled one or more times). Government grants promote lower recycling rates and high waste volumes because the material market price does not change and remains as high as before any public intervention. Therefore, recyclers perceive a reasonable profit with less effort suggesting that subsidies to recycling, when implemented alone, should stay at a moderate level.

Closer inspection of Fig. 4a shows that when subsidies and consumption taxes are applied separately, recycling rates are lower than those obtained from a tax-subsidy scheme. One reason is that a consumer tax alone curbs demand but does not provide direct incentives to recycle. Another reason is that, with only the recycling subsidy in place, recyclers’ profits are positively affected but not as much as when they are relieved from paying a landfill tax because the second-best recycling subsidy is not very high. However, when lithium demand decreases because of a consumption tax, subsidy levels can be increased, leading to higher recycling rates.

#### Demand and Waste

We turn now to analyze the accumulated effects of policy measures in the first 20 years. Figure 4b shows the total material demand ($y$), and waste ($w$) among policy scenarios. As mentioned above, a landfill tax reduces raw material prices, implying a material demand increase. However, despite the lower material price, demand also decreases because a positive landfill tax makes the waste price negative, which means that recyclers will not be willing to buy waste materials, and instead, consumers must pay to dispose of their waste. Likewise, consumer taxes increase purchasing material prices and depress material demand and waste.

Figure 4b highlights that a subsidy for recycling boosts waste and material demand. Recall that a recycling subsidy increases material prices (Eq. (2c) above) and waste prices (Eq. (3a) above), meaning that recyclers will be willing to buy waste as they benefit from higher material prices. As a result, consumers buy more materials and produce more waste. However, if governments combine recycling subsidies and consumption taxes with non-negative net government revenue constraints, the total cumulative demand and waste will be much lower than the benchmark scenario, and the policy will deliver later second-best results.

#### Raw Material Extraction and Recycling

We now evaluate how market price policies affect recycling and raw material supply. Figure 4c shows the total demand composed of raw and recycled materials. Extractive firms only receive incentives via market prices. As mentioned above, a landfill tax lowers material prices, reducing incentives to explore and extract raw materials. Recyclers still benefit from low but positive material prices and will process waste material to satisfy demand. The lithium market price also decreases after the government introduces consumption taxes, but consumers’ purchase price indirectly increases via the added costs of disposing of the material waste. Hence, production and consumption are slightly depressed.

As we pointed out (“Demand and Waste” above), recycling subsidies increase material and waste prices. Due to higher prices, raw material extraction will be slightly higher during the first 20 years. Compared to the benchmark, the recycled output will increase due to higher waste prices. With higher raw material extraction and recycled material, total resource demand will be relatively high, with only small welfare gains and damage reduction (see Fig. 4d). In addition, a tax-subsidy scheme depresses raw material extraction and stimulates recycling, but the effects are not as large as with the landfill tax.

#### Welfare Gains and Damage Reduction

The differences in cumulative welfare gains and damage reduction relative to the benchmark are shown in Fig. 4d. Among market-based instruments, a landfill tax offers the most damage reductions and welfare gains because higher waste disposal costs make recycling more attractive. Therefore, a landfill tax can prevent products from being disposed of prematurely and orient waste collection towards recycling.

As shown in Fig. 4d, positive social benefits will also occur if the government implements a tax on consumers as an advance disposal fee. However, with recycling only as an option, such a tax has little effect on recycling and waste reduction. As a result, welfare gains and damage reduction resulting from consumer taxes are very marginal compared to a first-best landfill tax. In addition, subsidies to recycling are ineffective because subsidies alone stimulate too much material demand. The benefits in welfare gains and damage reduction are better when combining subsidies with a consumption tax. However, the tax-subsidy scheme requires zero net government revenues each year. The second-best tax helps keep consumption from being too high, and the second-best subsidies are higher than in the scenario with only subsidies.

The results in this chapter suggest that the recycling efficient level depends not only on the marginal disposal cost but also on profit conditions that rely on market price levels. The following section, therefore, moves on to test the model validity and robustness of the optimal solutions.

### Sensitivity Analysis I: Non-market-Based Policies

This section elaborates a sensitivity analysis allowing decision-makers and modelers to select assumptions, as it illustrates how our model can accommodate different real-world situations. Table 3 describes three simulation scenarios. The first scenario involves government regulations limiting battery diversity and making more homogenous products, which reduces recycling costs. We double the *iota* ($\iota$) parameter which represents the recyclability levels in this scenario.^21^ In the second scenario, technological advances can lower recycling costs over time. To illustrate that situation, we increase the parameter *kapa* ($\kappa$) from 0.005 to 0.02, implying that recycling costs decrease by 2% instead of 0.5% per year.^22^ In the third scenario, a policy can lengthen a product’s lifespan to reduce waste production. In our model, the *gamma* ($\gamma )$ parameter is halved, implying a double battery lifetime.^23^ As a rule, improved recyclability, lower recycling costs, and extending the battery’s lifetime by investing in technology and product design typically come with a cost, which we do not incorporate in our model. Therefore, these welfare results need to be interpreted with caution.

**Table 3:** Simulation scenarios and parameter changes

Simulation scenario	Description	Symbol
Recyclability	An exogenous increase in the parameter $\iota$ reduces recycling costs	$\iota$ increases from 1 to 2
Technological change	An exogenous increase in the parameter $\kappa$ reduces recycling costs	$\kappa$ increases from 0.05 to 0.02
Longer lifetime	An exogenous decrease in the parameter $\gamma$ increases the lifetime	See Appendix Tables 12 and 13

**Fig. 5:**
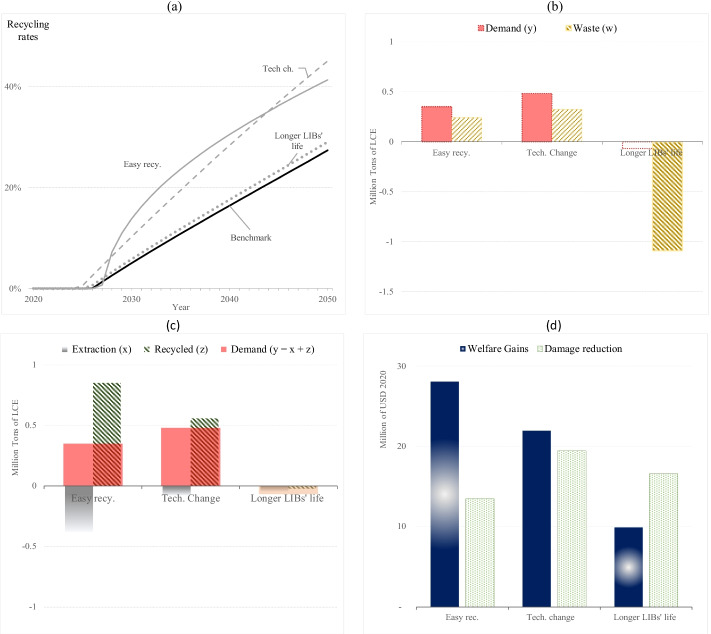
**a** Recycling rates among scenarios: easy recyclability, technological changes, longer LIB’s life. **b** The accumulated demand and waste (2021–2040) among scenarios compared to the benchmark. **c** The raw and recycled material production (accumulated until 2040) compared to the benchmark. **d** The welfare gains and damage reduction compared to the benchmark. Here, we calculate welfare and damages over a 100-year full-time horizon

Figure 5a shows that technological change and better product design also stimulate recycling. However, the effects are less immediate than in the landfill tax or recycling subsidy scenarios (Fig. 4a). In our model, technological change takes time (by assumption) and better product design to extend battery longevity slightly decreases marginal recycling costs.

Figure 5b shows that when recycling costs diminish because of higher recyclability or improved technologies, more recycled output is available to consumers reducing material prices. As a result, total material demand increases jointly with more waste creation. Therefore, recycling rates will be higher than a free market solution but similar to recycling rate levels resulting from a subsidy policy, as presented in Fig. 4a. In addition, Fig. 5b shows that longer battery life can extend material use and decrease material demand and waste vastly. Therefore, material circularity happens even if recycling rates are relatively low because longer battery life prevents waste accumulation.

Figure 5c illustrates that easy product recycling and technological change will lessen recyclers’ costs and put more recycled output in consumers’ hands. Therefore, material market prices decrease, and raw material supply reduces compared to the benchmark. It is essential to approach this account with caution because we do not include the cost of increasing recyclability as this model does not consider the battery production sector.^24^

Overall, welfare gains and damage reduction occur by extending the product lifetime or reducing recycling costs via better product design to easy recyclability and technological innovations. However, such measures *à la carrot* are not as effective as tax mechanisms to correct market prices and disincentivize waste production: *the stick*.

### Sensitivity Analysis II: Lower and Higher Damage Costs

This section performs a second sensitivity analysis to investigate how the optimal solution changes as damage costs change. In theory, landfill taxes should fully reflect the harmful waste cost. However, with limited data and research on the impact of electronic and battery waste, the costs of toxic waste damage are difficult to measure [37–39]. Therefore, in this study, we apply an approximate cost and the damage cost varies linearly with the amount of waste to simplify the model.

**Fig. 6:**
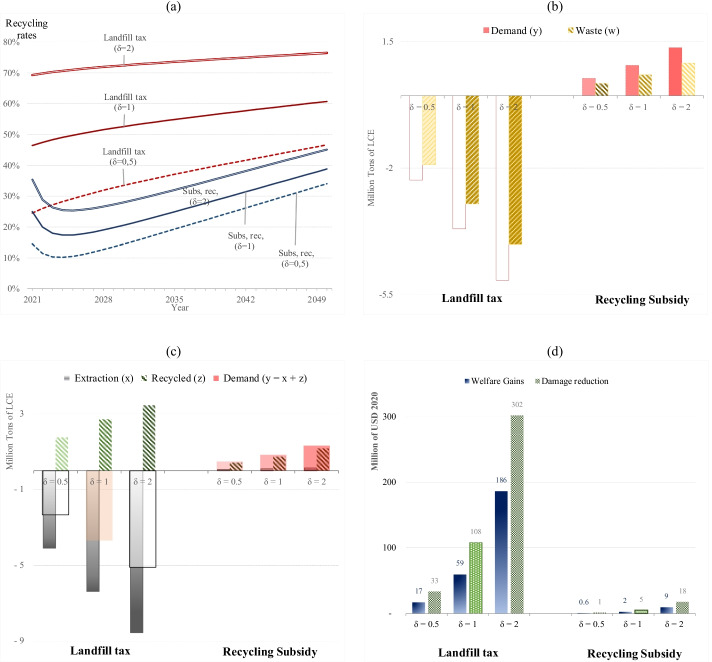
**a** Recycling rates among scenarios with different values for the damage parameter delta (δ) . **b** The accumulated demand and waste (2021–2040) among scenarios compared to the benchmark, i.e., free/unregulated market solution. **c** The raw and recycled material production (accumulated until 2040) compared to the benchmark. **d** The welfare gains and damage reduction compared to the benchmark. Here, we calculate welfare and damages over a 100-year full-time horizon

Figure 6a shows that higher damage costs imply higher recycling rates in response to higher landfill taxes. In the baseline scenario, the damage parameter delta is *δ* = 1. Figure 6b reveals that when we reduce the damage levels and half this parameter (*δ* = 0.5), cumulative demand and waste decrease 45% and 46%, respectively. By contrast, doubling damage levels (*δ* = 2) implies that cumulative demand and waste will be 32% higher than the benchmark scenario (*δ* = 1). Not surprisingly, the greater is the damage level, the lower is the effect of landfill taxes in terms of demand and waste reduction, and the sensitivity analysis suggests that the size of the damages has substantial impacts on the optimal level of material used.

Irrespective of damage levels, landfill taxes continue to reduce material extraction, and subsidies to recyclers continue to generate rebound effects, i.e., stimulating raw material extraction (Fig. 6c). Nonetheless, the greater is the damage level, the greater is the effect of landfill taxes on damage reduction and welfare gains (Fig. 6d).

Since we do not include externalities for raw material extraction, we do not apply Pigouvian taxes to the mining industry. However, the effect of a landfill tax on market prices is so pervasive that it reduces raw material supply and thus will also reduce externalities of raw material extraction.

## Discussion and Policy Implementation Challenges

In reviewing the literature, no empirical evidence was found to understand the cumulative effects of waste management policies on welfare and damage reduction considering budget requirements. An initial objective of this study was to provide a quantitative analysis that supplements previous literature on economic policies for material reuse. We show that accounting for harmful waste impacts is necessary to attain efficient recycling levels. Our simulations showed that a landfill tax is a first-best policy because it attacks the externality directly, stimulates recycling, and reduces toxic waste from spent batteries while welfare reaches its highest level. This finding is consistent with that of Hoogmartens et al. [17], who found that by applying a constant landfill tax, it is possible to approximate the first-best welfare optimal outcome very closely in terms of externality costs and lower raw material exhaustion.

One unanticipated finding was that after a social planner introduces a landfill tax, total material demand is lowest among all alternatives and scenarios because landfill taxes depress demand for raw materials and deviate it to recycled materials. Lower material use is undesirable if it implies slower green energy and digital transitions. Although our model may not fully represent the welfare benefits and positive externalities from battery use, we show that even if a landfill tax reduces material demand, society still perceives welfare gains because recycling attains much higher levels than without market interventions. Therefore, in response to the sustainability challenge, it would be advantageous if battery producers could use less material per product while maintaining their performance level, and the landfill tax gives incentives for that.

Another important finding was the ambiguous relationship between material extraction and recycling. In the case of abundant ore resources, extraction increases, and raw material prices fall, which lowers the incentive for recycling because recycling is too costly and immature compared to low-cost, mature mining. However, it is not straightforward how recycling affects raw material extraction. When implementing a landfill tax, raw material extraction will be lower than in a free market, and more recycled materials will satisfy demand. By contrast, if recyclers benefit via subsidies, such policy can lead to more raw material extraction, suggesting that the subsidy policy benefits are relatively moderate. This is an example of a phenomenon known as the rebound effect [40].^25^

In our model, a rebound occurs because subsidies to recyclers increase their demand for waste, increasing its price and reducing the cost of using materials, resulting in higher material consumption. Later, higher demand for materials leads to higher raw material prices, stimulating an initial raw material extraction. However, encouraging recycling in this way is not necessarily a welfare improver because the benefits in welfare gains and damage reduction are better if governments combine subsidies with a consumption tax. To some extent, the tax on consumers will dampen the subsidy rebound effect.

The parameter values and assumptions in this model are subject to uncertainty. With that in mind, we run a sensitivity analysis to investigate to what extent ambiguous information affects our results and conclusions, primarily related to the damage parameter. We demonstrated numerically that landfill taxes provide a consistent optimal solution with lower and higher damage levels. Since we do not include externalities of raw material extraction, we do not apply Pigouvian taxes to the mining industry. However, the effect of a landfill tax on market prices is so pervasive that it succeeds in reducing raw material extraction at all damage levels. By contrast, subsidies to recyclers continue to generate rebound effects by stimulating raw material extraction. Overall, this sensitivity analysis tests the robustness of the optimal solution and validates the model assumptions under poor information. However, policymakers should prioritize acquiring accurate data about damage levels to design more credible and proper policies.

In practice, an optimal market-based policy can bring counterfactual effects and implementation challenges. For example, charging waste holders directly for disposal costs may lead to illegal burning or dumping [41]. Likewise, implementing subsidies to recyclers may involve additional costs to monitor recycling firms’ activities, and recycling subsidies may create market distortions and more damage when illegal dumping is an option. In such cases, the subsidy should vary considering the monitoring costs, disposal costs, and recycling technologies, and deposit refunds are second-best [13]. Nonetheless, several questions remain unanswered about how to implement a combination of taxes (deposit) and subsidies (refund) when consumers and recyclers have different geographical locations and uncontrolled transboundary waste movements exist.

This paper stresses the use of circular reuse to minimize e-waste and create added value from used materials. Therefore, the study is unable to capture all externalities at different stages of materials’ life cycles associated with raw material extraction, recycling processes, and landfill pollution; it focuses only on the externalities of end-of-life products. However, avoiding the harmful effects of the entire life material cycle is crucial for a circular economy, so this is also an essential part of how policymakers should think about material circularity. Indeed, there are negative externalities from mining, and researchers have alerted that mining lithium can spoil unique landscapes and drain scarce water stocks [42]. In that case, implementing a Pigouvian tax on extraction, reflecting these environmental damages, would likely dampen raw material extraction, leading to higher market prices, stimulating recycling, and indirectly reducing landfill damages, too. Moreover, certain recycling processes can cause more harm than good [37], and the environmental benefits of recycling will depend on the recycling technology used and the material cocktails embedded in products [43–45]. Further research should be undertaken to integrate ecologically and carbon impacts across the material lifecycle and examine ways to make mining, recycling, and landfilling more sustainable.

## Conclusions

This research aimed to examine how a set of market-based policies can promote material circular reuse and correct market failures caused by improper waste disposal. The findings indicate that irrespective of damage levels, a landfill tax is the most efficient policy, as it targets the hidden cost of waste disposal and promotes the best results in recycling levels, damage reduction, and welfare gains. If a landfill tax is not feasible, other policies such as taxes, subsidies, and a tax-subsidy scheme provide second-best results. The research also shows that a consumer tax alone curbs demand but does not provide recycling incentives; thus, other market-based policies should be pursued.

This study has raised important questions regarding recycling subsidies. In general, a subsidy will encourage recycling. But if market prices do not reflect the externality cost, a subsidy to recyclers can promote material overuse because the subsidy will increase waste prices, which increases material value to consumers and leads to higher demand and waste. As the price of raw materials rises with higher demand, the initial raw material extraction is stimulated. Therefore, irrespective of damage levels, a high recycling subsidy cannot be the optimal policy because it increases waste demand and causes a rebound effect.

If governments want to avoid rebound effects, they should consider combining second-best policies. The numerical simulations confirmed that consumer taxes and recycling subsidies have limited welfare gains when implemented alone, while a tax-subsidy scheme will enhance welfare and reduce harmful waste with a neutral impact on the government’s budget. Subsidies alone will not be sufficient to curtail material demand and waste, and recycling will not reach optimal levels unless consumer taxes are applied with subsidies. That is why combining taxes and subsidies is more efficient than just one of the two policies.

Although this study focuses on the end-of-life externalities, the findings of combining economic policies may well have a bearing on the circular and sustainable use of materials. Notwithstanding the case of lithium, this work offers valuable insights into material and mineral markets, and the model framework can be applied with data of other critical raw materials. This research contributes to our understanding of why it may prove somewhat negligent to leave the market free and recycle adrift when society carries losses from harmful waste. The current findings support that recycling is essential for material circularity, but government intervention is required to moderate the material and recycling markets. By doing so, society can reap the benefits of reusing valuable materials and push forward sustainable energy and digital transitions.

## Data and Code Availability

The GAMS code and input data employed in this study are available upon request.

## Figures and Tables

**Fig. 7:**
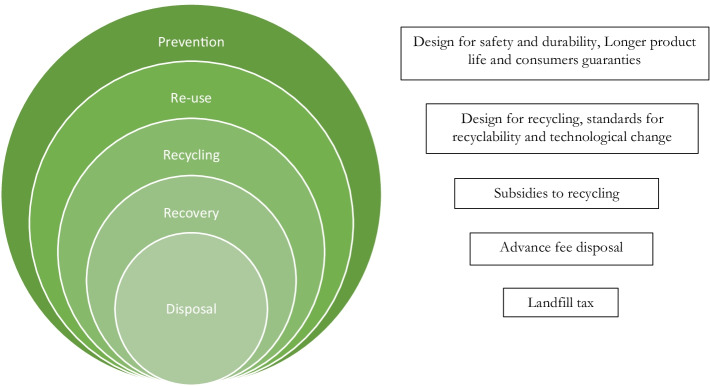
Policies and waste stock ladder based on the Waste Framework Directive [47]. This figure shows overlapping relationships in waste management and emphasizes waste escalation from less desirable outcomes such as landfill disposal to preferable preventive waste management. However, the accompanying policies in each ladder do not necessarily suggest a better efficient or optimal level

**Fig. 8:**
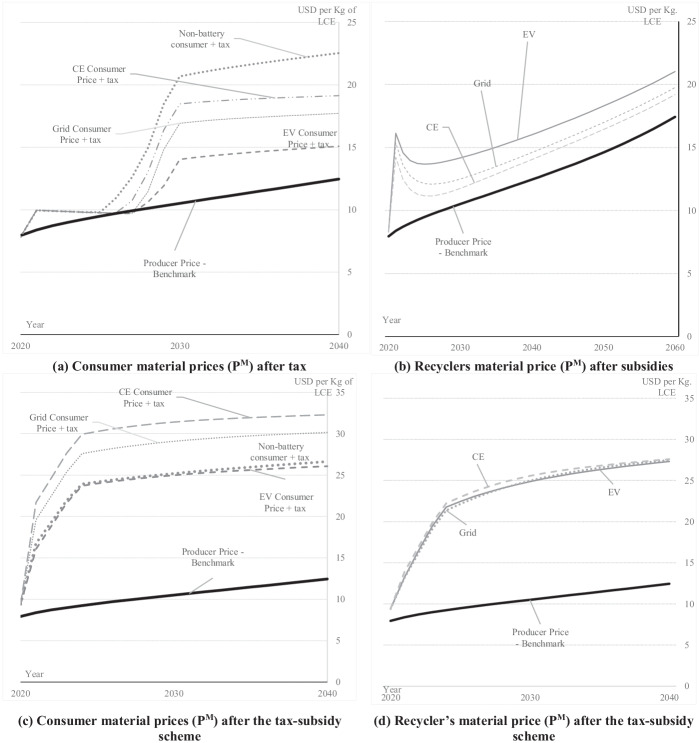
Material prices after taxes and subsidies are applied individually (**a** and **b**) or combined (**c** and **d**). Notice that consumer prices after taxes (**a**) increase much more than recyclers’ material prices after receiving a subsidy (**b**). However, both consumers’ and recyclers’ prices are much higher after governments implement a tax-subsidy scheme (**c** and **d**) compared to single tax and subsidy policies (**a** and **b**)

## Appendix 1. Demand function and data input

We assume the following utility function for the use of materials ${y}_{it}$ at each period “*t*” in all-consuming sectors “*i”*:

18 $${U}_{it}\left({y}_{it}\right)=\varsigma +{\beta }_{t}{\left({y}_{it}\right)}^{\frac{1+\epsilon }{\epsilon }}$$

where $\varsigma$ is some constant, $\epsilon$ represents the (long-term) price elasticity of demand, and ${\beta }_{t}=\frac{\epsilon }{1+\epsilon }{\left(\frac{1}{{y}_{0}{\sigma }_{t}}\right)}^{\frac{1}{\epsilon }}{p}_{0}$. The term ${y}_{0}$ denotes the initial demand level, while ${\sigma }_{t}$ is a function reflecting the underlying growth in demand. Plugging ${\beta }_{t}$ in (18):

19 $${U}_{it}\left({y}_{it}\right)=\varsigma +\frac{\epsilon }{1+\epsilon }{y}_{i0}{p}_{0}{\sigma }_{t}{\left(\frac{{y}_{it}}{{y}_{i0}{\sigma }_{t} }\right)}^{\frac{1+\epsilon }{\epsilon }}$$

Simplifying and making ${\varsigma }_{i}=0$

20 $${U}_{it}\left({y}_{it}\right)=\frac{\epsilon {y}_{it}{\left(\frac{{y}_{it}}{{y}_{i0}{\sigma }_{t} }\right)}^{\frac{1}{\epsilon }}{p}_{0}}{(\epsilon +1)}$$

This condition gives the following marginal utility function:

21 $${{U}_{it}}^{\mathrm{^{\prime}}}\left({y}_{it}\right)={\left(\frac{ {y}_{it}}{{y}_{i0}{\sigma }_{t}}\right)}^{\frac{1}{\epsilon }}{p}_{0}$$

Furthermore, the derived demand function, which is a price-dependent deterministic demand function that we use in the model numerical simulations, will take the following form:

22 $${y}_{it}-{y}_{i0}{\sigma }_{t}{\left(\frac{{p}_{t}}{{p}_{0}}\right)}^{\epsilon }\ge 0 \perp {y}_{it}\ge 0$$

The elasticity $\epsilon$ is a hypothetical value − 0.5 in the benchmark scenarios.^26^ We set the factor ${\sigma }_{t}$ from an exogenous growth function and calibrate the demand growth function ${\sigma }_{t}$ using several growth rates (see Table 4), and calibrated parameters (Table 5) following this functional form:

23 $${\sigma }_{it}=\frac{{\sigma }_{i1}}{{\sigma }_{i2}+{\sigma }_{i3}{e}^{-{\sigma }_{i4}t}+{\sigma }_{i5}{e}^{-{\sigma }_{i6}{t}^{2}}}$$

**Table 4:** The annual growth rate in lithium demand in sector *i* (given price in 2020)

Period	Transportation	Grid storage	Consumer electronics	Industrial applications
	(a)	(b)	(c)	(d)
Until 2025	25%	15%	10%	5%
2031–2050	8.5%	8.5%	3%	3%
2051–2100	3.5%	3.5%	1%	1%
After 2101	0%	0%	0%	0%

(a) Electric car registrations continue growing despite the pandemic. Meeting the 2030 target of the IEA and Paris Agreement implies that the global stock of electric cars should maintain annual growth rates above 25% by 2025 and in the range of 7 to 10% between 2030 and 2050 [36]

(b) Smart charging is crucial to ensure that grid capacity does not constrain electronic vehicle (EV) uptake [36]

(c and d) Growth rates until 2025 are extrapolations based on historical data [33]. The rate numbers from 2031 are our assumptions.

**Table 5:** Parameters in the demand growth function and displays the calibrated parameters of Eq. (23)

Parameter	Transportation	Grid storage	Consumer electronics	Industrial applications
${{\varvec{\sigma}}}_{{\varvec{i}}1}$	4982	2293	2636	2741
${{\varvec{\sigma}}}_{{\varvec{i}}2}$	6.07	6.41	295	489
${{\varvec{\sigma}}}_{{\varvec{i}}3}$	1113	1147	1229	2034
${{\varvec{\sigma}}}_{{\varvec{i}}4}$	0.074	0.072	0.053	0.053
${{\varvec{\sigma}}}_{{\varvec{i}}5}$	3863	1132	1112	218
${{\varvec{\sigma}}}_{{\varvec{i}}6}$	0.10	0.10	0.10	0.10

Lithium raw materials vary significantly in their lithium content, chemical compositions, and final use (Table 6). Our sources of information report mineral ore and reserves for hard rock and brine projects in different unit metrics, for example: in ppm Li, in percentages of Li, and in Li2O. In this paper, we used lithium carbonate equivalent (LCE). Since we took different information sources with other metric units, we normalized this data to “lithium carbonate equivalent” or “LCE” based on the table below’s conversion factors (Table 7). Lithium prices have fluctuated considerably over the last years (Table 8). In our simulations, we take 2020 as a base year.

**Table 6:** Demand for lithium in sector i (thousand tones — Kt — of lithium carbonate equivalent (LCE)). Source:[33, 36]

	Lithium in batteries from sales of EVs	Lithium in batteries from stocks of EVs	Grid storage	Consumers electronics (CE)	Non -battery use	Total
2015	7.80	24.2	0.9	60.2	103.3	172.2
2016	11.70	35.9	1.4	82.0	111.7	206.8
2017	16.90	52.8	4.1	185.8	162.4	369.2
2018	28.60	81.4	6.6	296.1	178.4	509.7
2019	32.50	113.9	6.9	285.7	132.8	457.8
2020e	34.92	109.1	7.4	267.6	126.6	436.5
Average		69.54	4.55	196.21	135.86	358.68

**Table 7:** Conversion factors for differing lithium data. Source: Savannah Resources

To convert from	Chemical abbreviation	To convert to:		
		Lithium (Li)	Lithium oxide (Li2O)	Lithium carbonate equivalent (LCE)
		Multiply by:		
Lithium	Li	1	2.153	5.323
Lithium oxide	Li2O	0.464	1	2.473
Lithium carbonate	Li2CO3	0.188	0.404	1
Lithium hydroxide monohydrate	LiOH.H20	0.165	0.356	0.880

**Table 8:** Price, annual average, battery grade of LCE in thousand USD per ton. Source: [33]

2014	2015	2016	2017	2018	2019	2020	Average 2014–2020
6.7	6.5	8.7	15.0	17.0	12.7	8.0	10.65

## Appendix 2. Supply data input (Tables 9 and 10)

**Table 9:** Estimated production, reserves and resources in 2020 per country in thousand tons. Source: [33]

	Production		Reserves (economically extractable)		Identify resources (technically feasible)	
	Li	LCE	Li	LCE	Li	LCE
Argentina	5.9	31.4	1.9	10.1	19.3	102.7
Australia	39.7	211.3	4.7	25	6.4	34.1
Bolivia	0	0	0	0	21	111.2
Chile	21.5	114.4	9.2	49	9.6	51.1
China	13.3	70.8	1.5	8	5.1	27.2
USA	Withheld	Withheld	0.8	4	7.9	42.1
Rest World	2.1	11.8	2.9	15.7	16.7	88.9
Total	82.5	439.2	21	111.8	86	457.8

**Table 10:** Initial unit extraction costs in thousand USD per ton of LCE

	Argentina	Australia	Bolivia	Chile	China	USA	Rest world	Average
Initial extraction cost* (USD/kg)	2.5	4.2^a^	6.0	3.6	5.24	3.4	6.6*	4.42 _§_
Transport cost${({\varvec{c}}}_{{\varvec{j}}}^{{\varvec{T}}})$(USD/kg)	0.4	0.67	0.96	0.58	0.84	0.54	1.05	0.8

*This value includes extraction and conversion costs. We compare average extraction cost from operating mines published by the Lithium Cost Model Service at Roskill and the Market Research at Deutsche Bank [34]

(a) Australia and the rest of the world produce spodumene lithium that needs to be refined into higher purity lithium products before being used in the battery supply chain. For example, China imports lithium concentrates and processes them in conversion plants

(§) Aritmetic mean value among countries

## Appendix 3. Damage cost estimation

Upon disposal, the two main hazards that LIBs pose are the high concentrations of leachable metals they contain and a tendency to explode and catch fire when improperly handled [38]. However, standards and regulations can improve the safety of electronic products and classify waste as hazardous and universal waste.

Deposited electronics in landfills release heavy metals like mercury, arsenic, lead, and heavy metals toxic for humans and ecosystems. Likewise, incinerating electronics releases heavy metals and other toxins into the air besides the typical greenhouse gas emissions.

Table 11 shows the economic cost of human exposure to harmful electronic waste. Waste generation and monetary damages vary between countries and regions. Waste production and economic damages are greater in Asia than in the rest of the world. Africa has the lowest waste generation, and economic costs are lower than other developing countries, but the impact on their economies can be devastating when considering those damage expenses as a percentage of GDP. Europe and North America have a relatively low economic cost of e-waste, but they head first globally regarding e-waste production per capita.

**Table 11:** E-waste generation and economic cost of health damages by country

	E-waste generation million tons (A)	E-waste generation per capita kg	From which batteries tons (B)	Annual economic lost (LEP) (billion USD) (C)	% GDP (D)	Economic cost of harmful waste batteries (thousand USD/ton) (B) * (C)
Africa	2.9	2.5	14,500	18	4%	6.21
North America (USA, Canada)	7.7	20.2	38,500	51	0.33%	6.62
Central and South America	5.4	8	27,000	33	2.04%	6.11
Eastern Asia (China, Japan, Korea)	13.7	9	68,500	227	1.80%	16.57
West, Central and South Asia	11.2	5	56,000	236	1.80%	21.07
Europe	12	16.2	60,000	13	0.31%	1.08
Oceania	0.7	16	3500	NA	NA	
**Total**	53.6		264,500			10.93

A. [4]

B. Eurostat Statistics (2018)

C. Neurodevelopmental damages assessed as decrements (or reductions) in intelligence quotient (I.Q.) points are the most evident impact of harmful e-waste in human health. This damage is translated into decreased lifetime earning potential, assessed as lost lifetime economic productivity (LEP). Estimations based on [4, 46]

D. [4] and Eurostat Statistics (2018)

## Appendix 4. Policy scenarios and waste stock ladder

Note that only disposal, recycling, and prevention are explicitly captured by our model (see Fig. 7), whereas we do not analyze material recovery and product reuse.^27^ “Market-Based Policies” explains the logic of market-based policies (landfill taxes, consumption taxes, and subsidies to recyclers). In this Appendix, we describe other regulatory measures exogenously defined, for which we ignore the cost of such actions.

### Design for Recycling and Technological Changes

In our numerical analyses, we apply the following formulation of recycling costs, accounting for technological innovations via exogenous cost reductions over time:

24 $${C}^{R}\left(\alpha \right)={cr}_{0}\left[1-\mathrm{ln}\left(1-{\alpha }^{\iota }\right)\right]{e}^{-\upkappa t}$$

The cost of the cheapest unit of recycled output $\left(z=\alpha w\right)$ is then ${cr}_{0}\bullet {e}^{-\upkappa t}.$ When $\alpha \to 1$, we see that the (marginal) costs go towards infinity, as required above. The marginal recycling cost functions will be:

25 $$\frac{d{C}^{R}\left(\alpha \right)\alpha w}{d\alpha }={{cr}_{0}e}^{-\upkappa t}\left[1-\mathrm{ln}\left(1-{a}^{\iota }\right)+\frac{\iota {a}^{\iota }}{1-{a}^{\iota }}\right]w$$

In the lithium context, policies may enforce standards to reduce the immense variability of battery designs and enforce more recyclable batteries. The parameter $\iota (iota)$ determines this level or ease of recyclability. The higher is this parameter; the slower marginal recycling costs will increase. The recycling cost function also includes technological progress that reduces the unit costs exogenously over time through the parameter $\kappa$. Thus, the measures we consider here involve exogenously increases in $\iota$ (recyclability) and $\kappa$ (technological change) (see Table 12).

### Design for Safety — Consumer Guarantees and Longer Product Lifetime (Gamma)

In the waste management hierarchy, prevention is the most desirable way to manage waste. Here, policies may promote extended consumer guarantees offering options to repair and replace their batteries without any additional charge.^28^

The parameters $\gamma$ used in Eqs. (5) and (6) denote the annual depreciation rate of material stocks in use (thus, 1/$\gamma$ is a measure of the resource lifetime before it must be recycled or discarded). In our numerical simulation, we change this parameter to extend the product lifetime and calculate the respective effects on waste, welfare, and damages.

**Table 12:** Policy scenarios and simulation parameters under free market and social planner solution

Scenario	Free market benchmark	Social solution
*Initial recycling costs** ${(cr}_{0})$	10 (thousand USD per ton)	10 (thousand USD per ton)
Recyclability** ($\iota :iota)$	$\iota$= 1	$\iota$= 2
Technological change** ($\kappa :kappa)$	$\kappa$= 0.005	$\kappa$= 0.02
Longer battery life	See table below	See table below

* This is the approximated cost of recycling 1-ton cathode materials from spent Li-ion batteries, including fixed and variable costs. It varies among cathode chemistries, recycling methods, and geographical location. Kushnir and Sanden [48﻿] estimate an approximately recycling cost of between 6 and 10 thousand USD per ton of cathode materials from spent Li-ion batteries; Wang et al. [24] observe that for an existing recycling facility, the variable costs can vary from USD 1100 to USD 4500 per ton of recovered materials. They also assess that when total costs equal total revenue at a breakeven point, the unit value of recovered materials varies between 890 and 8900 USD per ton depending on the type of cathode chemistry. In the future, that cost may fall due to the increasing volume of collected EV. Li-ion batteries and advancements in recycling technologies

**Hypothetical values

**Table 13:** Battery lifetime in years and depreciation rate of lithium-ion batteries by sector. Own assumptions

	Electric vehicles		Grids		Consumer electronics	
	Years	Depreciation rate	Years	Depreciation rate	Years	Depreciation rate
Short lifetime	10	0.10	5	0.20	3	0.33
Long lifetime	20	0.05	10	0.10	6	0.17

## Footnotes

1 Take-back programs such as “Extended Producer Responsibility” encourage product weight reduction, product life extension, and warranty extension on repairs. In Europe, these measures have been updated to a new circular economy action plan [9].

2 Lithium is one of the 30 critical raw materials found in e-waste presenting supply risks and difficult recycling [20]. Until now, data and information about lithium material stocks and flows are the most reliable and publicly accessible. See Appendices.

3 The effects of market power and strategic behavior have been studied extensively in previous studies (c.f. [21, 22]) concluding that a monopolistic industry will slow down extraction vis-à-vis the socially optimal solution when facing a potential recycler. Hoogmartens et al. [17] extend the analysis of strategic behavior to the possibility of a substitute material at a fixed price.

4 Illegal disposal and transboundary waste shipment are real but beyond the scope of this study. Ino [23] offers a framework to analyze how to prevent firms from disposing waste illegally.

5 In our numerical simulations, we only consider lithium recycling from LIBs. Cobalt and nickel, however, can also be recycled simultaneously. In lab-scale recycling experiments, recycling efficiencies vary between materials (cobalt 89%), nickel 69%, and lithium 80%) [24]. For simplicity, we do not differentiate recycling efficiencies by technology (i.e., hydrometallurgical, pyrometallurgical, or direct recycling); nor do we differentiate recycling efficiency based on input scrap type or quality.

6 Economies of scale are important at the initial stages of recycling business and recycling profits dependent greatly on the composition of the total scrap stream [24].

7 Time index *t* is suppressed where it is not essential.

8 Allowing consumers to deliver the material waste directly to the landfill, paying the price {P}^{LF}, would not change the outcome of our analysis (assuming they are not able to dump waste outside the landfill).

9 Quasi-linear preferences are useful for isolating one sector and avoiding income effect feedback on the demand for other goods [26]. Quasi-linear utilities also make the externality optimal level independent of the consumers’ wealth [27]. See Appendix 1.

10 $\dot{M}$ means $\frac{dM}{dt}$, and the subscripts other than *t* denote the respective partial derivatives.

11 This is a *rising supply cost* case, in which the marginal cost rises as the cheaper sources are depleted [28].

12 The quality of ore may change in case of high-quality resource scarcity, but it is not observed at the moment. Nevertheless, our simulation accounts for cost differentiation due to the necessity of additional processes, transport costs, and the costs of readjusting output from the base year level to the optimal level (see Table 10in Appendix 2).

13 As accumulated production imposes a constraint on the remaining profits, the costate variable (﻿{﻿\lambda }^{﻿E}﻿) would otherwise be negative.

14 As extraction costs vary with ore grades, it is logical to deplete the cheapest resource first [29]. Once low-cost resources become exhausted, extraction turns towards deeper and costlier deposits. While extraction costs increase, scarcity rents may or may not decrease with time [30].

15 In our model, landfill capacity is large enough to accommodate the recycling residues. However, the modelling framework can be extended to incorporate a landfill capacity constraint: ${{S}_{\mathrm{LF}}}_{t}\le \overline{{S }_{\mathrm{LF}}}$ as suggested by Hoogmartens et al. [17].

16 It is generally accepted that social discount rate should be lower than the private one [14, 17, 31].

17 See Appendix 4.

18 During 2020 and 2021, most industries experienced widespread supply chain disruptions due mostly to COVID-19 according to the USGS [33].

19 To date, about 25% of identified lithium resources are economically feasible (so-called reserves). Nonetheless, identified lithium resources have doubled during the last 5 years going from 41 to 86 million tons [33]. In addition, new exploration projects grow in large numbers and will put more than 200 million tons of lithium resources available to the market within the coming years [34]. There are also economic concentrations of minerals, metals, and rare materials in the deep oceans, adding to the identify resource stock and sustainability challenge [35].

20 See Appendix 5 (Fig. 8) for additional results discriminated by consumer sector.

21 See the Appendix 4, and Eq. (24).

22 See the Appendix 4, and Eq. (25).

23 See the Appendix 4, and Tables 12 and 13.

24 Battery costs have fallen and will continue falling markedly [36]. This sector is constantly working on reducing material content in batteries while optimizing performance. This may increase battery recyclability and extend battery lifetime.

25 This rebound effect resembles the Jevons paradox; i.e., a more efficient resource use will accelerate resource exhaustion. This effect also shares similarities with the green paradox as pointed to by, e.g., [15]. The green paradox suggests that environmental policies may lead to accelerated extraction of fossil fuels, especially if fuel owners believe the policy to be strengthened over time. Although the mechanism is somewhat different in our model, we find that policies to increase circularity may in some instances accelerate extraction of raw materials.
